# Cancer Mortality Trends in Spain (2000–2016): Differences between Immigrant and Native Populations

**DOI:** 10.3390/ijerph17145127

**Published:** 2020-07-16

**Authors:** Adriana Oliva-Arocas, Pamela Pereyra-Zamora, José M. Copete, Andreu Nolasco

**Affiliations:** Research Unit for the Analysis of Mortality and Health Statistics, Department of Community Nursing, Preventive Medicine, Public Health and History of Science, University of Alicante, 03080 Alicante, Spain; adriana.oliva@ua.es (A.O.-A.); copetealacant@yahoo.co.uk (J.M.C.); nolasco@ua.es (A.N.)

**Keywords:** mortality, emigration and immigration, neoplasms, trends, Spain

## Abstract

Spain’s population has changed thanks to recent immigration. Therefore, a new epidemiological and demographic profile has been generated in the country. This study aims to analyze immigrant and native cancer mortality trends in Spain for the period 2000 to 2016. An ecological study of trends was carried out. Age-standardized rates of cancer mortality (ASR) and annual percentage change (APC) between groups and study sub-periods were calculated. Significant decreases in ASR were observed for cancer in both the native and the immigrant populations, in both men and women. However, in 2014–2016, there was an increase in ASR in the immigrant population compared to 2011–2013, due to the increase in ASR among immigrants from European regions. Differences in ASR by cancer between immigrant and native populations residing in Spain have been identified, both in the rate of decline and magnitude as well as by the birth region of the immigrant population. The increase observed in the cancer mortality trend at the end of the period in some immigrant groups indicates the need to monitor these indicators given the demographic, social, and economic changes.

## 1. Introduction

During the last few decades, migratory patterns have become more diverse and complex, motivated by causes such as poverty, inequality, violence, conflict (armed, ethnic, environmental, etc.), and the search for employment [1]. In countries like Spain, its attraction as a retirement destination is also an important incentive [2,3]. According to the United Nations (UN), the number of immigrants worldwide reached a figure of 258 million in 2017. Of these, more than 30% resided in Asia (30.9%), the continent with the highest number of immigrants, followed by the European region (30.2%) and North America (22.4%) [1]. For some years, the immigrant population residing in the European Union (including the United Kingdom) has shown a relatively stable increase [4]. In 2015, some 54.4 million immigrants (10.7%) resided in the EU, placing Spain in fourth place with 5.9 million [5].

Although Spain is a country with a short immigration history, there has been a significant increase in the number of immigrants since the beginning of this century. Thus, it has become one of the countries with the highest proportion of immigrants in the world. In 2016, the immigrant population residing in Spain represented 13.2% of the total population [6]. According to some authors, migratory flows have produced a series of demographic and epidemiological changes, both in the country of origin and in the host country. Changes in mortality patterns have been particularly relevant, with cancer being one of the main causes of death [7,8,9].

According to data from the National Statistics Institute (INE), tumors have been the primary cause of death among men (35.9%) in Spain in 2016 and the secondary cause of death among women, at a rate far higher than diseases of the circulatory system (21.6%) as regards the total number of deaths in that year [10]. Regarding cancer mortality trends, several studies carried out in different countries have shown that there is great variability in outcomes, mainly due to the different types of cancer. Downward trends in cancer mortality have been observed for decades in some countries of Asia, North America, and Europe. However, the pace and magnitude of this reduction have been uneven among the different countries. In some of them, some stability in mortality rates has been observed, whereas in others, this reduction has nevertheless increased [11,12,13,14,15,16]. Although some countries have an important track record in immigration studies, the trends and patterns of mortality among the native and immigrant populations have seldom been analyzed and compared. Studies of cancer mortality are even scarcer, despite evidence of differences in mortality rates between both populations [7,8,17]. In addition, the existence of favorable cancer mortality indicators among the immigrant population as compared to the native population [18,19] is also known.

Over the last few years, several important demographic changes have occurred in Spain; i.e., until 2009, immigration to Spain was higher than emigration, but from 2010 to 2016, a change in the migratory pattern occurred, with emigration being higher than immigration. Nevertheless, since 2017 until today, immigration has again become the main trend [20]. Moreover, the arrival of the 2008 economic crisis propelled social and economic changes that in 2012 led to cuts in healthcare and in the right to access it (RD-L 16/2012) [21]. Nevertheless, little is known as yet about the effects of the crisis and the cutbacks in the healthcare system on the immigrant population residing in the country, but the impact on their mortality might be noticeable in the forthcoming years. As far as we know, no studies on the mortality trends for all types of cancer in both the native and immigrant populations, that would allow comparisons, have been carried out. Furthermore, since the study period for this research is long, it will allow the observation of changes in mortality trends and patterns in Spain.

In summary, this study aims to analyze cancer mortality trends in the immigrant and native populations residing in the Spanish state in 2000 to 2016.

## 2. Materials and Methods

### 2.1. Data Source

This is an ecological study of mortality trends, for which anonymized data of residents’ deaths in Spain between 2000 and 2016 have been used. This data was obtained from the Statistical Death Bulletin by request to the National Institute of Statistics (INE), and the figures of the resident population between 2000 and 2016 by age and sex were obtained from the Municipal Register of Inhabitants and disseminated by the INE. As these are administrative data obtained retrospectively, the approval of an ethical committee is not necessary in Spain.

Immigrant status was established, taking into account the place of birth (in both data sources) and classifying the population as native or immigrant (those born outside the Spanish territory). The immigrant population was classified according to the region of the country of origin: Eastern Europe, Northern Europe, Western Europe, Southern Europe, Africa, America, Asia, and Oceania. Finally, it was decided not to take Oceania into account in the analysis as the number of deaths by cancer during the study period was very low. Mortality data were obtained by age, sex, place of birth, and cause of death for each of the years studied. The cause of death was then classified under the tenth International Statistical Classification of Diseases (ICD-10); the causes of Chapter II—Tumors (neoplasms): C00-D48 [22] have specifically been considered.

### 2.2. Analytical Methodology

Mortality rates were calculated by sex for the native and immigrant populations (and for the total in both populations). They were also calculated for any type of cancer and standardized by age (ASR) through the direct method, using the 2013 European population as the standard population [23]. Rates were calculated as a whole and separated by geographical region of origin, and the corresponding confidence intervals were also calculated at 95% (95% CI). For the calculation of the ASR, the age ranges considered were 0, 1–4, 5–9, until 85 and over.

Two different strategies were also considered for the analysis of trends and their changes. On the one hand, the annual trends and the ASR inflection points were analyzed throughout the entire period using Joinpoint regression models for each sex and origin (native/immigrant) as well as for all specific regions of birth. For each identified trend segment, the annual percentage change (APC) of the ASR was calculated with its corresponding CI 95%. A negative APC will indicate a downward trend, while a positive APC indicates an increase in the ASR trend. The Joinpoint Regression Program v4.6.0.0 [24] was used to construct the model and trend graphs. Secondly, in order to provide greater stability to the data, the entire analysis period has been divided into six study sub-periods: 2000–2001, 2002–2004, 2005–2007, 2008–2010, 2011–2013, and 2014–2016. The percentage change in the ASR between the last sub-period (2014–2016) and the rest of the sub-periods of analysis were calculated.

In addition, graphs depicting trends and disaggregated by population groups (total, native, and immigrant) and for each of the sub-periods were also made in order to observe the evolution of cancer mortality indicators. Moreover, a level of *p* < 0.05 was also considered to determine the significance of the results.

Finally, the proportional mortality by sex and specific cause of cancer were calculated to determine the main causes in each of the population groups. The Excel^®^ program and the SPSS^®^ statistical program have also been used to calculate the indicators.

## 3. Results

**Figure 1:**
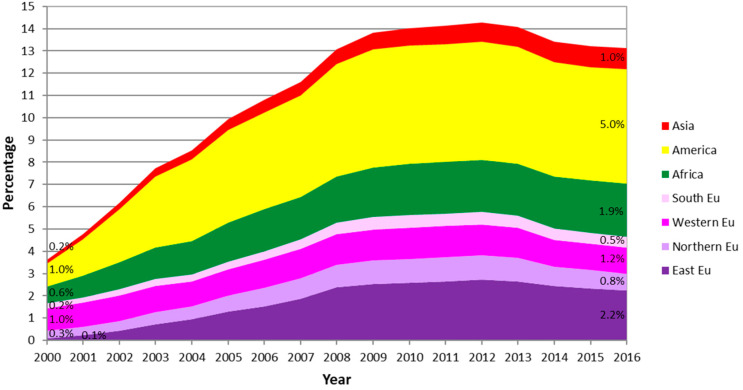
Evolution of the immigrant population of both sexes in Spain according to region of birth for the period 2000–2016.

The population resident in Spain has increased from 40,499,791 in 2000 to 46,557,008 in 2016. In 2000, the immigrant population was 1,472,459 (3.6%), and it was 6,123,769 (13.2%) in 2016; the increase during this period was +316%. This growth in the immigrant population shows differences according to the region of birth (Figure 1). The regions of origin that experienced the greatest growth were Eastern Europe (with an increase of +3161% between 2000 and 2016), Asia (+534%), America (+478%), and Africa (+258%).

**Figure 2:**
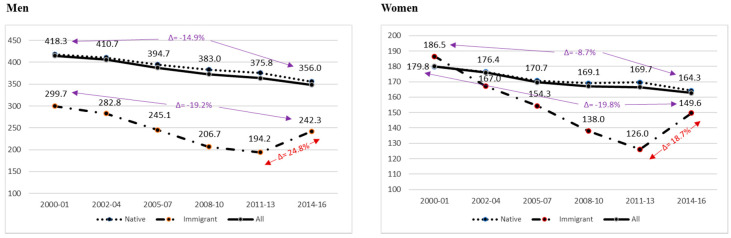
Age-standardized rates of cancer mortality (ASR) per 100,000 people in the immigrant, native, and total populations by sex and sub-period (2000–2016).

During the study period, there was a total of 1,772,468 deaths from cancer in both sexes, 3.5% of which correspond to immigrants. When analyzing the evolution of ASR among natives and immigrants over time, it can be observed that the native population (in both sexes) has higher ASR than those of the entire immigrant population throughout the study period, as well as a clear trend of mortality due to descending cancer. Nevertheless, these declines were more pronounced in the case of men. However, there is a marked downward trend in the immigrant population (in both sexes), more pronounced than that of the natives, until the 2011–2013 period, and a shift in the trend at the end of the study period (see Figure 2). This increase in ASR in the immigrant population during the 2014–16 sub-period was significant as compared to the previous 2011–2013 sub-period in both men and women, reaching an increase of 24.8% in men and 18.7% in women. It should be noted that despite this reverse shift of the cancer mortality trend in the immigrant population, the decrease in ASR at the beginning of the period (2000–2001) is still significant, reaching a reduction of approximately 20% for each sex (see Table 1).

When analyzing the ASR disaggregated by region of origin (see Table 1), in men, it can be observed that all immigrant groups had lower rates of cancer mortality throughout the period vis-à-vis the native population. In the case of women, those from Asia, America, Africa, and the South and East of Europe had higher ASR at the beginning of the period, but this reduced over time. Depending on the region of origin, the majority of immigrant groups experienced a significant decrease in mortality rates concerning the first sub-period, except for men from Eastern, Western, and Southern Europe and women from Eastern, Northern, and Western Europe. This relative decrease observed in ASR in immigrant men ranges from 19.5% (Northern Europe) to 64.8% (Asia), showing also significant declines in those from Africa and America. In the case of women, the decrease ranged from 28.3% in those from America to 64.1% in the Asian population. Those from Africa and Southern Europe also showed significant declines in ASR. When analyzing the shift in the cancer mortality trend of the last period (2014–2016) as compared to 2011–2013 according to the region of origin, a significant increase has been observed in the four European regions in both men and women (except for immigrants from Southern Europe).

**Figure 3:**
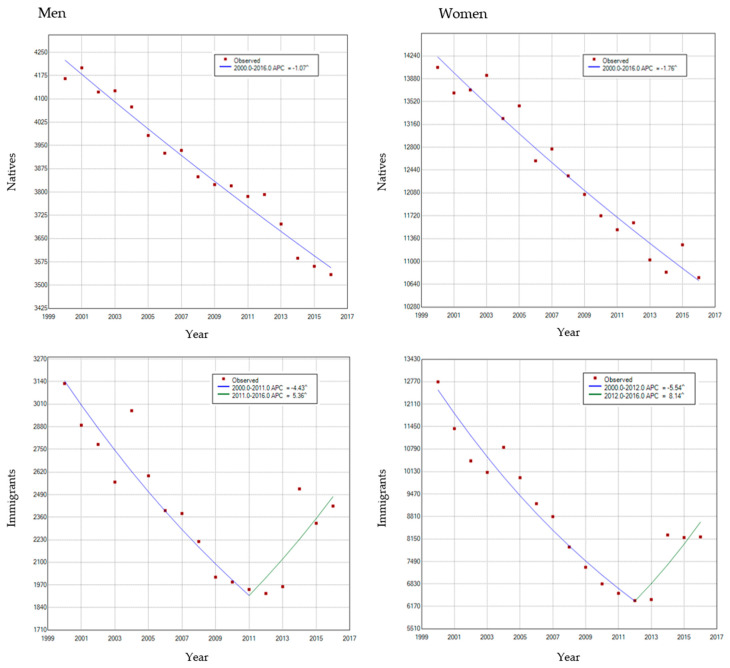
Age-standardized rates of cancer mortality (ASR), Joinpoint, and trends in immigrant and native populations in both sexes, for the period 2000–2016.

**Figure 4:**
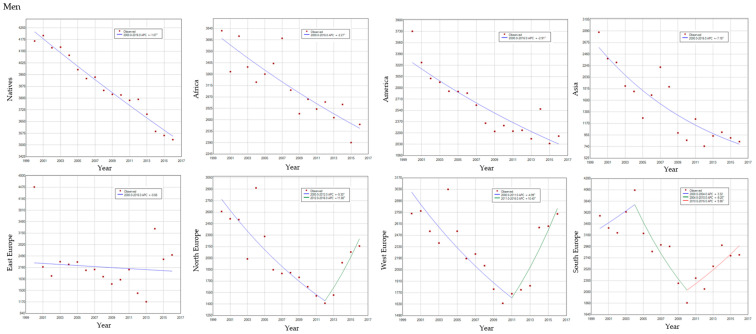

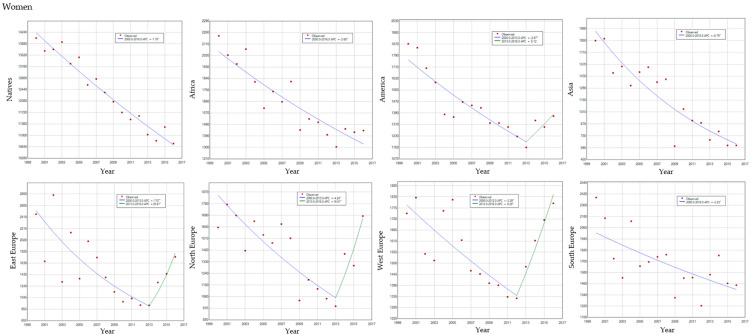
Age-standardized rates of cancer mortality (ASR), Joinpoint, and trends in immigrants by region of birth and natives in both sexes, for the period 2000–2016.

Figure 3 and Figure 4 show the significant changes in the annual trend of cancer mortality in the immigrant population in both sexes, while in the native population, there is a progressive decrease throughout the entire period of analysis. In Figure 3, the significant increase in cancer mortality in the immigrant population is noticeable. This increase has occurred since the year 2011 in men and since 2013 in the case of women. In Figure 4, it can also be seen that in the case of immigrant men, the shift in the trend of cancer mortality was particularly due to the increase in ASR in men from the Northern European regions (+11.8%), the Western (+10.4%), and the Southern (+5.9%). As regards women, this increase can also be observed in those from the European region (East + 25.7%, North + 16.6%, and West + 8.3%) and America (+5.1%) respectively.

**Figure 5:**
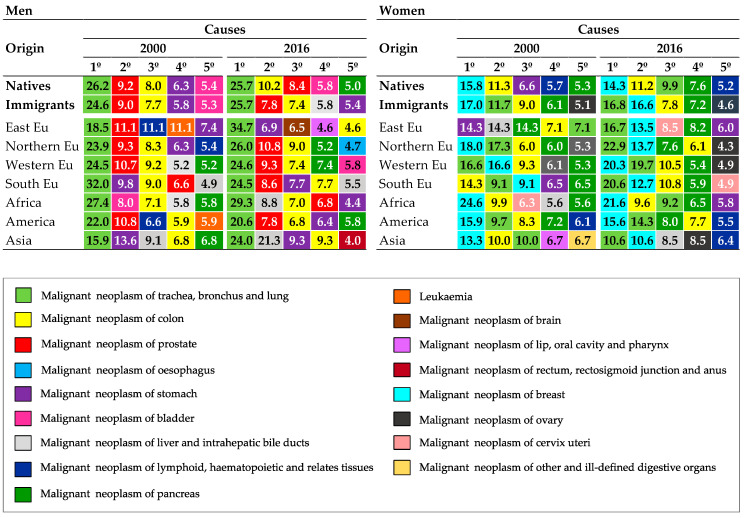
Proportional mortality, five main causes of death by type of cancer in men and women (natives and immigrants) according to region of birth, in 2000 and 2016.

The pictogram of Figure 5 contains information on the proportional mortality by type of cancer. It shows that malignant tumors of the trachea, bronchi and lung, prostate (in men), breast (in women), and colon, the first five causes of death from cancer, remain as the main causes of death throughout the period in both native and immigrant populations and for both sexes. It is worth noting the rapid rise in the percentage of deaths caused by malignant tumors of the trachea, bronchial tubes, and lungs in native women, which moves from the seventh position in the year 2000 to the third since the year 2010 to 2018. Likewise, an increase in deaths by malignant pancreas tumors in women, both native and immigrant, can be observed during the study period.

## 4. Discussion

### 4.1. Main Findings

This study reveals a clear downward trend in cancer mortality in Spain over 16 years (from 2000 to 2016) in both the native and immigrant populations, although an uptick has been observed in the immigrant population over the last few years. Differences have been found in both the magnitude and the rhythm of the ASR decrease between these two population groups, particularly by sex and region of birth. Although studies on cancer mortality among the immigrant population are scarce, the results found in this study are consistent with those observed in most countries of the European Union [25]. There is evidence that in some countries with a long history of immigration, the immigrant population, in general, has a lower mortality pattern than the native population. This pattern has been observed both in general mortality, in cancer mortality, and in some specific causes of cancer [7,9,26,27,28,29].

Due to the great diversity of backgrounds of the immigrant population residing in Spain, it is difficult to identify an explanation for the observed results. On the one hand, the causes could stem from the countries of origin themselves, and on the other, particularly in some specific causes of cancer, they could be found in the host country. Hence, the shift in the cancer mortality trend observed since 2011 in the immigrant population (particularly in immigrants from Europe as a whole) could reflect the epidemiological and mortality patterns observed in some European countries. Despite the achievements in reducing cancer mortality in most of these countries in some of the eastern and south-eastern regions of Europe, high rates of cancer mortality in both sexes are still found as compared to Europe as a whole [25,30,31,32]. Countries like Hungary and Slovakia, for example, have the highest rates of cancer mortality in both sexes in the entire continent. On the contrary, as in the case of some countries in Northern Europe, such as Latvia, Estonia, and Lithuania (regarding men), and in Southern Europe (Croatia and Serbia in both sexes), Western Europe is the region with the lowest cancer mortality rates [26].

The slowdown in the decrease rhythm of ASR for cancer and the subsequent increase observed in the immigrant population could also be related to acculturation processes and the length of stay of the immigrant population in the host country. Assimilation and adaptation to the lifestyles of the host country have been widely described. This would lead to a convergence with the native population in mortality patterns that might have become visible over the last few years [33,34,35,36]. At the end of the 20th century, during the 1980s and 1990s, there was an increase in international immigration in Spain, motivated by political changes and economic growth, that gave way to a situation of prosperity in the country. This immigration flow was motivated both by work reasons and by retirement, led by the European population that decided to stay permanently in Spain [17,37], among other factors.

The social and economic changes which occurred in Spain at the onset of the economic crisis in Europe (2008) are also an important element to take into account in the analysis [38]. The austerity measures adopted by European countries reduced public spending, privatized public services, and deregulated the market. Consequently, health inequalities in the younger population, immigrants, and ethnic minorities increased [39].

In relation to the significant decreases in ASR for cancer in the Asian population throughout the study period (both men and women), this phenomenon might be consistent with the trends in cancer mortality observed in some Asian countries, where reductions have been observed during the last two decades. In countries such as Japan, there is a downward mortality trend, thanks in large part to the decrease in death rates from stomach, liver, and gallbladder cancer in both sexes [14]. In addition, in Korea, there has been a great increase in cancer survival for all cancer types in both men and women [16].

On the other hand, trends in cancer mortality have been observed to be decreasing both in North America and in Latin America, an observation that is coincident with our data. Canada and the United States, for instance, show decreases in cancer mortality during the last 30 years, although within the US, the pattern was more heterogeneous [13,40]. In the case of Latin America, there is a great diversity of trends among the different countries. Countries such as Mexico and Chile have also shown decreasing trends in cancer mortality during the last decade; however, countries such as Argentina and Brazil show stability in their mortality rates due to the increase in mortality from some types of cancer [41,42].

Regarding the results found in the population from Africa, other research, such as that of Moncho et al. [7] and Pereyra-Zamora [17], has found that until 2008, the population was already experiencing significant decreases in general mortality and cancer in Spain. The reason might be that this immigrant group is still young and therefore they maintain the healthy immigrant effect.

### 4.2. Methodological Strengths and Limitations

The sources of information used in this study do not collect information about the year of the arrival of the immigrant population or the length of stay in Spain. Studies on mortality and immigration could contain various biases, such as salmon bias, numerator/denominator bias, or the so-called “healthy immigrant effect” described by Pereyra-Zamora [17]. The bias that could justify this mortality advantage of the immigrant population present in our results is the “effect of the healthy immigrant”, which confers lower mortality rates in the immigrant population, maintaining lower figures than the native population of the developed countries to which they emigrate [43].

The present study is one of the first to analyze the trend of cancer mortality in Spain among both the native and the immigrant populations for a prolonged period. All the analyses have been carried out using the country of origin as a variable. This has allowed the inclusion of origin into the analysis, regardless of possible changes in nationality.

## 5. Conclusions

In conclusion, this study has shown differences in cancer mortality in Spain between immigrant and native populations, both in the rate of decline and magnitude, as well as in the sex and region of birth of the immigrant population. The change in trend detected in some immigrant groups indicates the need to conduct detailed studies on possible explanatory variables for this process, as well as to carry out analyses by specific causes. Finally, monitoring these health outcomes could contribute to the implementation of social health policies aimed at maintaining these downward trends in specific and highly vulnerable populations, as is the case of the immigrant population.

## Figures and Tables

**Table 1:** Age-standardized rates of cancer mortality (ASR) per 100,000 people and percentages of change between immigrant and native populations for men and women by region of birth (2000–2016).

**Men**																								
**Origin**	**SP1 = 2000-01**			**SP2 = 2002-04**				**SP3 = 2005-07**				**SP4 = 2008-10**				**SP5 = 2011-13**					**SP6 = 2014-16**			
	**Deaths**	**ASR**	**95%CI**	**Deaths**	**ASR**	**95%CI**	**∆% SP2 vs. SP1**	**Deaths**	**ASR**	**95%CI**	**∆% SP3 vs. SP2**	**Deaths**	**ASR**	**95%CI**	**∆% SP4 vs. SP3**	**Deaths**	**ASR**	**95%CI**	**∆% SP5 vs. SP4**	**∆% SP6 vs. SP5**	**Deaths**	**ASR**	**95%CI**	**∆% SP6 vs. SP1**
**Natives**	117,552	418.3	415.9–420.8	181,130	410.7	408.8–412.7	−1.8	183,830	394.7	392.9–396.6	−3.9	188,330	383.0	381.3–384.8	−3.0	195,030	375.8	374.1–377.5	−1.9	−5.3	194,711	356.0	354.4–357.6	−14.9
**Immigrants**	2580	299.7	287.4–312.1	4898	282.8	274.0–291.6	−5.7	5672	245.1	237.6–252.6	−13.3	6611	206.7	200.9–212.5	−15.7 *	7581	194.2	189.2–199.1	−6.1 *	24.8 *	8434	242.3	236.2–248.4	−19.2 *
**East Eu**	44	306.4	191.5–421.3	111	228.0	161.8–294.2	−25.6	235	221.0	163.2–278.7	−3.1	420	179.0	140.1–218.0	−19.0	548	153.6	124.1–183.1	−14.2	77.7	736	272.9	227.8–318.0	−10.9
**Northern Eu**	443	255.6	230.7–280.5	956	252.3	235.4–269.3	−1.3	1115	194.5	181.6–207.3	−22.9 *	1440	172.1	162.3–181.9	−11.5	1558	148.0	140.1–155.8	−14.0 *	39.0 *	1608	205.7	194.4–217.0	−19.5 *
**Western Eu**	706	275.5	253.6–297.4	1293	272.2	256.1–288.2	−1.2	1276	230.5	216.6–244.3	−15.3	1376	183.7	173.4–194.0	−20.3	1571	180.5	171.2–189.8	−1.7	45.6	1702	262.8	249.6–276.0	−4.6
**South Eu**	241	343.2	298.1–388.3	448	369.4	333.4–405.4	7.6	467	301.7	271.9–331.5	−18.3 *	471	233.1	209.6–256.5	−22.7 *	549	235.6	214.1–257.1	1.1	20.9 *	641	284.7	260.5–308.9	−17.0
**Africa**	462	354.0	313.5–394.6	863	345.6	315.6–375.6	−2.4	1075	346.5	318.6–374.5	0.3	1075	292.0	268.3–315.7	−15.7	1253	280.7	260.5–300.9	−3.9	−6.6	1362	262.3	244.1–280.4	−25.9
**America**	599	347.0	317.7–376.3	1080	294.3	274.8–313.8	−15.2 *	1339	274.6	257.1–292.1	−6.7	1460	229.6	214.8–244.3	−16.4 *	1543	219.1	205.5–232.6	−4.6	2.5	1801	224.6	211.7–237.5	−35.3 *
**Asia**	82	259.5	192.3–326.8	143	201.5	158.6–244.3	−22.4	155	173.9	135.0–212.9	−13.7	151	119.8	91.3–148.4	−31.1	154	96.9	74.0–199.7	−19.2	−5.7	186	91.4	70.9–111.8	−64.8
**Women**																								
**Origin**	**SP1 = 2000-01**			**SP2 = 2002-04**				**SP3 = 2005-07**				**SP4 = 2008-10**				**SP5 = 2011-13**					**SP6 = 2014-16**			
	**Deaths**	**ASR**	**95%CI**	**Deaths**	**ASR**	**95%CI**	**∆%SP2 vs. SP1**	**Deaths**	**ASR**	**95%CI**	**∆%SP3 vs. SP2**	**Deaths**	**ASR**	**95%CI**	**∆%SP4 vs. SP3**	**Deaths**	**ASR**	**95%CI**	**∆%SP5 vs. SP4**	**∆%SP6 vs. SP5**	**Deaths**	**ASR**	**95%CI**	**∆%SP6 vs. SP1**
**Natives**	70,231	179.8	178.5–181.2	107,925	176.4	175.4–177.5	−1.9	110,196	170.7	169.6–171.7	−3.3	115,338	169.1	168.1–170.1	−0.9	122,164	169.7	168.7–170.7	0.4	−3.2	123,831	164.3	163.3–165.2	−8.7
**Immigrants**	1969	186.5	178.0–195.0	3598	167.0	161.2–172.9	−10.4 *	4478	154.3	149.3–159.3	−7.6 *	5375	138.0	133.8–142.2	−10.6 *	5878	126.0	122.4–129.7	−8.7 *	18.7 *	6839	149.6	145.6–153.6	−19.8 *
**East Eu**	31	204.8	125.4–284.1	124	205.1	153.1–257.1	0.1	249	170.4	131.2–209.5	−16.9	377	114.2	90.4–138.0	−33.0	510	95.2	79.6–110.8	−16.6	52.6	707	145.3	124.2–166.3	−29.1
**Northern Eu**	296	169.4	149.3–189.5	568	162.8	148.5–177.2	−3.9	812	154.9	142.9–166.8	−4.9	941	123.0	114.4–131.7	−20.6 *	1015	104.7	97.8–111.6	−14.9 *	38.8 *	1082	145.3	135.6–154.9	−14.2
**Western Eu**	542	178.9	163.2–194.6	908	167.0	155.7–178.4	−6.6	1041	161.9	151.6–172.2	−3.1	1159	141.4	133.0–149.8	−12.7	1273	138.2	130.4–146.0	−2.3	23.7	1333	171.0	161.5–180.5	−4.4
**South Eu**	153	227.2	190.3–264.1	223	181.1	156.6–205.7	−20.3	253	169.9	148.1–191.7	−6.2	287	152.8	134.4–171.3	−10.0	293	143.6	126.6–160.6	−6.0	8.4	321	155.7	138.1–173.3	−31.5 *
**Africa**	298	210.3	183.5–237.0	530	197.4	178.2–216.6	−6.1	554	167.0	151.0–183.0	−15.4	650	158.1	144.2–172.1	−5.3	670	139.7	127.7–151.7	−11.7	2.4	835	143.0	132.0–154.0	−32.0
**America**	589	185.7	169.8–201.6	1132	156.1	146.1–166.1	−15.9 *	1425	142.3	133.8–150.9	−8.8	1692	135.4	127.6–143.1	−4.9	1750	122.2	115.3–129.2	−9.7	9.0	2134	133.2	126.3–140.0	−28.3 *
**Asia**	59	185.6	133.0–238.2	107	149.2	115.9–182.6	−19.6	132	146.4	114.4–178.4	−1.9	115	100.2	76.5–123.9	−31.6	117	82.4	63.3–101.5	−17.7	−19.2	133	66.6	50.7–82.5	−64.1

Abbreviations: ASR: Age-standardized mortality rates, ∆%: Percentage change, * Statistically significant (*p* < 0.05), 95%CI: Confidence interval.
